# Elucidating carbohydrate preference and engineering glucose transport in *Caldimonas thermodepolymerans* for enhanced polyhydroxyalkanoate production

**DOI:** 10.1007/s00253-026-13741-0

**Published:** 2026-02-13

**Authors:** Xenie Hajkova, Anastasia Grybchuk-Ieremenko, Pavel Dvorak, Iva Buchtikova, Vojtech Cerny, Viktorie Chvatalova, Stanislav Obruca

**Affiliations:** 1 Faculty of Chemistry, Brno University of Technology, Purkynova 118, 612 00 Brno, Czech Republic; 2 Department of Experimental Biology (Section of Microbiology, Microbial Bioengineering Laboratory), Faculty of Science, Masaryk University, Kamenice 5/E25, 625 00 Brno, Czech Republic

**Keywords:** *Caldimonas thermodepolymerans*, Polyhydroxyalkanoates, Thermophiles, Sugar metabolism, Glucose transporters, Lignocelluloses

## Abstract

**Abstract:**

*Caldimonas thermodepolymerans* DSM 15344, a moderately thermophilic bacterium, has emerged as a promising candidate for next-generation industrial biotechnology (NGIB) due to its ability to utilize lignocellulose-derived sugars for polyhydroxyalkanoate (PHA) production. This study assesses its metabolic potential by evaluating the utilization of various plant-derived sugars and their mixtures, with a focus on xylose, glucose, and cellobiose. The results indicate that *C. thermodepolymerans* exhibits a strong preference for xylose (3.97 g/L PHB) over glucose (2.28 g/L PHB) but demonstrates even greater efficiency in metabolizing cellobiose (4.96 g/L PHB). However, extracellular hydrolysis of cellobiose leads to glucose accumulation, which constrains overall productivity. Our findings suggest that the primary limitation in glucose metabolism is inefficient glucose transport rather than intracellular catabolism. To address this bottleneck, the *glf* glucose facilitator gene from the mesophilic bacterium *Zymomonas mobilis* was introduced into *C. thermodepolymerans*, enhancing its glucose utilization capacity. The engineered strain (Cald_GLF3) exhibited significantly improved PHA productivity, particularly when cultivated on sugar mixtures containing cellobiose. Despite being grown at suboptimal temperatures due to the thermal instability of Glf from *Z. mobilis*, Cald_GLF3 outperformed the wild-type strain, achieving notably high PHA yields when cultivated with cellobiose as the sole carbon source (9.26 g/L PHB). These findings highlight the critical role of glucose transport in the metabolism of *C. thermodepolymerans* and suggest that targeted engineering can further enhance its biotechnological potential. This study establishes *C. thermodepolymerans* as a promising thermophilic chassis for PHA production from lignocellulosic sugars, contributing to sustainable biopolymer synthesis.

**Key points:**

- *C. thermodepolymerans DSM 15344 produces PHA from lignocellulose-derived sugars*
- *Xylose and cellobiose are preferred substrates, while glucose is poorly utilized *
- *Deficient glucose transport in DSM 15344 restored by Zymomonas mobilis glf gene*

## Introduction

Modern human society faces a myriad of challenges, and among the most pivotal, unavoidable, and formidable is the ongoing shift of the chemical industry from reliance on fossil resources to renewable alternatives. Microbial biotechnologies stand poised to play an important role in facilitating this transition. Microorganisms exhibit the remarkable ability to utilize various renewable resources and transform them into a diverse array of valuable products, encompassing fuels, chemicals, and materials (Jerome et al. 2022; Gawel et al. 2019).

Microbial biotechnologies can also contribute to solution of “plastic crisis” (Borrelle et al. 2020) by providing alternatives to petrochemical polymers. Annual global plastic waste generation exceeds 350 million tonnes, and the life cycle of plastics is associated with greenhouse gas emissions of approximately 2 gigatonnes of CO_2_ equivalents per year (OECD 2022; Plastics Europe 2024). Beyond climate impacts, plastic pollution poses a well-documented threat to aquatic and terrestrial organisms through physical damage, chemical toxicity, and bioaccumulation across food webs (National Academies of Sciences, Engineering, and Medicine 2022). Together, these quantitative and ecological impacts provide the broader motivation for investigating biodegradable plastic alternatives. Polyhydroxyalkanoates (PHA) represent microbial polyesters that various prokaryotes accumulate as storage and stress robustness enhancing metabolites (Obruca et al. 2022). As eco-friendly alternatives, PHA surpass synthetic polymers in sustainability and environmental impact (Fu et al. 2023; Rajvanshi et al. 2023). Regarding environmental fate, PHA are readily biodegradable under many biologically active conditions: industrial composting test report ≥ 90% mineralization within 6 months (50–70 °C) and freshwater tests have shown rapid mineralization in weeks to months under favorable conditions. By contrast, conventional polyethylene, polypropylene, or polyethylene terephthalate typically persists for decades to centuries in natural environments (Koller et al. 2025).

Nevertheless, traditional microbial technologies exhibit several weaknesses which are manifested in high investment and operational costs for these processes. In response to these challenges, Chen and Jiang (2018) have recently introduced the concept of next-generation industrial biotechnology (NGIB). This innovative approach relies on employing extremophiles as chassis for bioproduction. The inherent resilience of extremophiles to extreme conditions endows these processes with natural robustness against microbial contamination (Yu et al. 2019). Apart from cultivation, the extremophilic nature of employed microorganisms provides benefits in upstream processing; for instance, the salt tolerance of some extremophilic microorganisms allows the simple integration of an acidic/alkaline hydrolysis step in the processing of complex substrates such as lignocelluloses (Kucera et al. 2018). Furthermore, it may also facilitate streamlined downstream processing as demonstrated in the isolation of intracellular products from hypotonic lysis–sensitive cells (Novackova et al. 2022).

To establish the NGIB process, identifying the optimal extremophilic microbial host is paramount. The desirable microorganism should exhibit resilience, stability, and the ability to efficiently utilize cost-effective renewable resources such as lignocelluloses. A high-level understanding of its genetic and metabolic features is essential for further improvement of the bacterium employing synthetic biology tools and approaches. Currently, within the family of halophiles, the moderately halophilic bacterium *Halomonas bluephagenesis* stands out as a promising candidate for manufacturing not only PHA but other high-value products as well (Park et al. 2023; Zhang et al. 2023).

However, the utilization of thermophiles—microorganisms adapted to high temperatures—offers a set of advantages by introducing extreme conditions solely through elevated temperature. Unlike with halophiles, challenges such as salt-related costs and corrosion are absent. Contrary to common misconceptions about the energy demands of thermophilic biotechnologies, well-isolated bioreactors require relatively low energy for heating to moderate thermophilic temperatures. Additionally, thermophilic cultivations can function as “self-heating systems” due to the heat energy generated by microbial metabolism and dissipated through stirring, particularly in industrial-scale cultivations with high cell densities. Furthermore, thermophilic processes exhibit high energy efficiency, as only minimal energy-demanding cooling efforts are necessary (Turner et al. 2007; Ibrahim and Steinbüchel  2010) .

*Caldimonas thermodepolymerans* DSM 15344 (formerly known as *Schlegelella thermodepolymerans*) is a gram-negative, moderately thermophilic, aerobic bacterium, emerging as a promising candidate for a thermophilic chassis in NGIB processes. This bacterium was initially isolated from activated sludge collected in Fayoum, Egypt by Elbanna et al. (2003) due to its notable ability to degrade various polyesters, as reflected in its taxonomic name. Despite this capability being investigated in subsequent studies (Romen et al. 2004; Elbanna et al. 2004), the bacterium received limited attention for an extended period. However, our recent findings demonstrated that *C. thermodepolymerans* not only degrades PHA, but it can also produce them in high quantities, and it shows preference for xylose. These characteristics are not only intriguing from a fundamental perspective but also position *C. thermodepolymerans* as a robust candidate for NGIB processes and industrial PHA production, especially from diverse lignocellulose-based resources (Kourilova et al. 2020). Lignocellulosic biomass is a complex heterogeneous plant material composed primarily of cellulose, hemicellulose, and lignin. Hemicellulose contains various pentose and hexose sugars, including xylose, while cellulose represents a major source of glucose, with additional carbohydrates such as cellobiose and xylobiose formed during hydrolysis (Segers et al. 2024). The genomic features and some of the metabolic characteristics of this bacterium have been recently elucidated (Musilova et al. 2021; 2023). The bacterium was evaluated as a potent candidate for PHA production from xylose-rich hemicellulose-based resources and demonstrated its proficiency in xylose metabolism and tolerance to lignocellulose-relevant microbial inhibitors such as phenolic compounds or derivatives of furfural (Kourilova et al. 2021). In a study, Zhou et al. (2023) delved into cultivation parameters influencing PHA synthesis in *C. thermodepolymerans* and employed proteomics to unravel metabolic pathways for PHA synthesis from xylose in this bacterium. Recently, *C. thermodepolymerans* demonstrated the ability to achieve high cell densities and elevated PHA productivity on xylose in laboratory bioreactors using a fed-batch cultivation strategy under nitrogen-limited conditions (Jang et al. 2025). The capability of *C. thermodepolymerans* with respect to direct utilization of xylan was also reported (Zhou et al. 2025). *C. thermodepolymerans* was also utilized for PHA biosynthesis from wine lees, side products of prosecco wine production (Caminiti et al. 2025). In the immediate past, an initial genome-editing toolkit was developed for *C. thermodepolymerans*, potentially opening numerous opportunities for its utilization as a thermophilic chassis for bioproduction (Grybchuk-Ieremenko et al. 2025).

A detailed understanding of metabolic features is a crucial step in the development of extremophilic chassis. Musilova et al. (2023) proposed a comprehensive scheme of the central carbohydrate metabolism of three related *Caldimonas* strains, including *C. thermodepolymerans* DSM 15344. However, the range of plant-derived sugars—both monomers and oligomers—and their mixtures that *C. thermodepolymerans* can metabolize and efficiently convert into PHA has not yet been thoroughly investigated. Furthermore, other critical metabolic aspects of *C. thermodepolymerans*, such as the kinetics of sugar utilization and potential bottlenecks in the efficient processing of certain sugars, remain to be elucidated. A deeper understanding of these fundamental characteristics could be crucial in establishing *C. thermodepolymerans* as a promising thermophilic chassis in the NGIB framework.

The objective of this study was to assess various plant-derived sugars and their mixtures as potential substrates for PHA production by *C. thermodepolymerans*. We investigated the utilization kinetics of xylose, glucose, cellobiose, and their combinations, alongside studying microbial culture growth and PHA biosynthesis parameters. Additionally, we addressed the deficiency in glucose metabolism within *C. thermodepolymerans* by introducing a high-capacity glucose transporter from *Zymomonas mobilis*. Our findings suggest that the limitation in glucose metabolism in *C. thermodepolymerans* is primarily attributed to glucose transportation into bacterial cells rather than a lack of metabolic capacity for glucose utilization. These results underscore the significant potential of *C. thermodepolymerans* as a microbial platform for PHA production within the framework of NGIB. Furthermore, our study demonstrates that its biotechnological potential can be further enhanced through metabolic engineering.

## Materials and methods

### Strains and cultivation media

The microorganism used in this study is a bacterial strain *C. thermodepolymerans* DSM 15344 from DSMZ-German Collection of Microorganisms and Cell Cultures. Bacterial suspensions were stored in cryotubes with 10% glycerol as cryoprotectant in a deep freezer at −80 °C. Bacteria were cultivated in two types of media. First, nutrient-rich complex medium Nutrient Broth w/w 1% Peptone (HiMedia, India) was used—50 mL in 100-mL Erlenmeyer flasks.

The basic mineral salt medium (MSM) with the following composition was used as production medium—Na_2_HPO_4_ · 12 H_2_O (9.0 g/L), KH_2_PO_4_ (1.5 g/L), NH_4_Cl (1.0 g/L), MgSO_4_ · 7 H_2_O (0.2 g/L), CaCl_2_ · 2 H_2_O (0.02 g/L), Fe^(III)^NH_4_citrate (0.0012 g/L), yeast extract (0.5 g/L), 1 mL/L of microelements solution (EDTA (50.0 g/L), FeCl_3_ · 6 H_2_O (13.8 g/L), ZnCl_2_ (0.84 g/L), CuCl_2_ · 2 H_2_O (0.13 g/L), CoCl_2_ · 6 H_2_O (0.1 g/L), MnCl_2_ · 6 H_2_O (0.016 g/L), H_3_BO_3_ (0.1 g/L), dissolved in distilled water) and the specific type and concentration of the carbon source (usually 20 g/L). Based on the medium composition, the MSM imposed nitrogen-limited conditions, with an estimated C/N ratio ranging from approximately 25:1 to 31.5:1 depending on the carbon source applied. Such nitrogen limitation is known to promote intracellular PHA accumulation and was therefore intentionally employed during the production phase.

### Integration of the glucose transporter into *C. thermodepolymerans* genome

The *glf* gene from *Z. mobilis* (NCBI gene ID 79904430), which codes for the glucose facilitator protein, was integrated into the chromosome of *C. thermodepolymerans* strain DSM 15344. The mini-Tn5 transposon vector pBAMD1-6_*glf* constructed previously (Bujdoš et al. 2023) was electroporated into *C. thermodepolymerans* competent cells.

To prepare electrocompetent bacterial cells, the overnight culture of *C. thermodepolymerans* was inoculated into 100 mL of fresh LB medium (Serva) to the OD_600_ of 0.05 and shaken at 220 rpm at 37 °C. Upon reaching an OD_600_ of ~ 0.5, the bacterial culture was cooled on ice and washed three times with 10% glycerol in milli-Q water. Cells were then resuspended in 1.5 mL of ice-cold 10% glycerol, aliquoted, and frozen at −60 °C (Grybchuk-Ieremenko et al. 2025). Ten electroporations with pBAMD1-6_*glf* vector were conducted in parallel. For this purpose, 100 µL of cells was thawed on ice, and 100 ng of plasmid DNA was added. Electroporation was performed using a Biorad Gene Pulser Xcell (2500 V, 25 μF, 200 Ω) with a 2-mm-gap cuvette. Immediately following the pulse, 0.9 mL of prewarmed (37 °C) LB was added to the cells, which were then recovered and shaken at 220 rpm at 37 °C for 2 h. Afterward, bacterial cultures were transferred into 10 mL of LB (in 50-mL Erlenmeyer flasks) supplied with 10 µg/mL gentamicin and cultured at 42 °C, 220 rpm for 24 h. Cultivation was performed at 42 °C, a temperature previously identified as a suitable compromise for *C. thermodepolymerans* (Grybchuk-Ieremenko et al. 2025); this setting maintains a high growth rate near the 50 °C optimum while preserving the functional integrity of the mesophilic Glf transporter. Subsequently, isolates were passaged twice every 24 h in 10 mL of MSM medium with 5 g/L glucose and 10 µg/mL gentamicin at 42 °C, 220 rpm. Then the ODs were measured, and the isolate that showed the fastest growth on glucose was chosen for further examination. It was spread for single colonies on MSM agar plates supplied with 5 g/L glucose and 10 µg/mL gentamicin and incubated at 42 °C. The streaking on fresh MSM plates with glucose was repeated several times to ensure the stability of the integration. The growth of all obtained mutants was tested in the presence of 5 g/L glucose also at *C. thermodepolymerans’* optimal temperature of 50 °C. Forty single colonies in two replicates obtained from preselection in liquid and solid media were tested for the growth in 600 µL of MSM with 2 g/L glucose in a 48-well plate in Infinite® 200 PRO plate reader (Tecan). The isolate Cald_GLF3, which showed repeatedly the fastest growth at 42 °C, was taken for further investigations and its growth was compared to the wild type in the 48-well plate assay under the conditions described above. To identify the locus of the *glf* integration in the chromosome, arbitrary PCR with standard primers as described by Martínez-García et al. (2014) was used.

### Screening of lignocellulose-derived sugar utilization potential of *C. thermodepolymerans*

Screening for carbohydrates occurring in lignocellulosic matrices was performed in 12-well plates for suspension culture. The prepared plates were incubated in the thermoshaker Biosan PST-60HL-4 at 50 °C with constant shaking (280 rpm). The volume of MSM including bacterial culture (5% v/v) was 2 mL per well in biological triplicates. The carbohydrates tested were xylose, xylobiose, glucose, cellobiose, cellotriose, maltose, and maltotriose. For control, cultivation without a carbohydrate source was also performed. Due to the high purchase cost of some of the tested carbohydrates, specifically cellotriose, maltotriose, and xylobiose, the substrate concentration was adjusted to 10 g/L. As a consequence of the lower substrate concentration, the cultivation time was reduced to 48 h. After this period, the samples for biomass and PHA analysis were collected. The selection of carbon sources was initially based on the study by Musilova et al. (2023), which reported distinct growth responses of the studied microorganism to xylose, glucose, and cellobiose. Based on these findings, the present study further extended the substrate spectrum to include structurally related disaccharides and oligosaccharides. Xylose, glucose, cellobiose, xylobiose and cellotriose represent typical products of lignocellulosic biomass hydrolysis, whereas maltose and maltotriose are not generated during this process but were deliberately included as glucose-derived structural analogues to facilitate comparison of microbial growth on related α- and β-linked carbohydrates.

### Kinetics of utilization of xylose, glucose, and cellobiose and their mixtures

The ability and efficiency of utilizing various carbohydrates (xylose, glucose, cellobiose) and their combinations for growth and PHA production were monitored using submerged cultivations in Erlenmeyer flasks in biological triplicates, with sampling every 12 h. The medium composition and cultivation protocol were based on section *Strains and cultivation media*. The inoculation phase lasted 20 h, after which 5 vol. % of the culture was transferred to the production medium (100 mL in 250-mL Erlenmeyer flasks) containing—in addition to MSM—20 g/L of a carbon source. The tested carbohydrates were added in equal proportions. In the case of two carbohydrates, each was present at 10 g/L. When xylose, glucose, and cellobiose were combined, the concentration of each carbohydrate was 6 g/L. Cultivation in MSM was terminated after 72 h. The biomass yield coefficient (*Y*_X/S_) and PHA yield coefficient (*Y*_P/S_) were calculated according to Eqs. (1) and (2). PHA content was calculated according to Eq. (3). The volumetric PHA concentration (P, g·L^−1^) was calculated from the biomass concentration and PHA content according to Eq. (4). Maximal volumetric biomass (*q*_Xmax_) and PHA (*q*_Pmax_) productivity, and substrate utilization rate (*q*_Smax_) were determined as the slopes of linear regressions fitted to the respective linear segments of biomass, PHA, and substrate profiles over time (Eqs. (5)–(7)).

1 $${Y}_{\mathrm{X}/\mathrm{S}}=\frac{\Delta X}{\Delta S}$$

2 $${Y}_{\mathrm{P}/\mathrm{S}}=\frac{\Delta P}{\Delta S}$$

3 $$\text{PHA content }(\mathrm{\%})=\frac{P}{X}\times 100$$

4 $$P=X\times \frac{\text{PHA content}}{100}$$

5 $${q}_{\mathrm{X},\text{ max}}=max\left(\frac{dX}{dt}\right)$$

6 $${q}_{\mathrm{P},\text{ max}}=max\left(\frac{dP}{dt}\right)$$

7 $${q}_{\mathrm{S},\text{ max}}=max\left(-\frac{dS}{dt}\right)$$

*X*, biomass (g·L^−1^); *P*, PHA (g·L^−1^); *S*, substrate (g·L^−1^); *t*, cultivation time (h).

### Analytical methods

Following sample collection at the respective cultivation time points specified above, biomass and culture supernatants were processed for further analyses. Dry biomass was determined gravimetrically. From each cultivation, performed in biological triplicates, 10 mL of culture was collected. The sample was then centrifuged, washed with distilled water, and centrifuged again. The resulting pellet was dried to a constant weight and subsequently weighed.

The content of PHA in dried biomass was analyzed by gas chromatography with a flame ionization detector, following the method described by Obruca et al. (2013). PHA content was determined as methyl esters of 3-hydroxy acids, formed by methanolysis of the intracellular polymer. Approximately 8–11 mg of dry biomass was reacted with 1 mL of chloroform and 0.8 mL of 15% sulfuric acid in methanol containing 5 mg/mL of benzoic acid as an internal standard. The reaction was carried out at 94 °C for 3 h. After completion, 0.5 mL of 0.05 M NaOH was added, and the mixture was vigorously shaken. Following phase separation, 0.05 mL of the organic phase was mixed with 0.9 mL of isopropyl alcohol. The resulting methyl esters were analyzed using a Trace GC Ultra (Thermo Fisher Scientific) equipped with a Stabilwax column (30 m × 0.32 mm × 0.5 µm) and a flame ionization detector. Calibration was performed using a commercial poly(3-hydroxybutyrate) (PHB) homopolymer standard. Since GC-FID responses depend on PHA monomer composition, individual monomer peaks were evaluated; however, only 3-hydroxybutyrate-derived peaks were detected in all samples, confirming PHB as the sole polymer produced.

The supernatants obtained during sampling were used for the analysis of carbohydrates present in the cultivation medium. Prior to measurement by high-performance liquid chromatography (HPLC) with a refractive index detector, the samples were filtered through a nylon membrane filter with a pore size of 0.45 µm. The analysis was performed using a Shimadzu LC-10AD HPLC system. For isocratic separation, a Water Carbohydrate Analysis column (3.9 × 300 mm) was used, with a mobile phase consisting of acetonitrile and water in a ratio of 80:20. Carbohydrate concentrations were calculated from peak areas using calibration curves for xylose, glucose, and cellobiose.

All experiments were performed in biological triplicates and results are presented as mean ± standard deviation. Statistical significance between the WT and engineered strains was evaluated using an unpaired two-tailed Student’s *t*-test, with *p* < 0.05 considered statistically significant.

## Results

### Utilization of lignocellulose-relevant sugars by *C. thermodepolymerans*

Plant biomass holds promise as a resource serving as a viable alternative to fossil resources. Therefore, we screened the capacity of *C. thermodepolymerans* to utilize various plant-relevant sugars including disaccharides and trisaccharides; the results are shown in Table 1. The bacterium exhibited a pronounced preference for xylose among the tested monosaccharides, surpassing its affinity for other sugars, including glucose. Our results indicate that the bacterium is unable to cleave glycosidic bonds between xyloses in xylobiose since we observed only negligible growth of the culture on this disaccharide (Table 1).

**Table 1:** Screening of catabolic potential of *C. thermodepolymerans* with respect to selected plant-derived sugars, cultivations were carried out in 12-well plates within 48 h time interval at 50 °C with initial sugar concentration 10 g/L

	OD 630 nm	CDW [g/L]	PHB (g/L)
Xylose	6.443 ± 1.025	2.933 ± 0.283	0.773 ± 0.019
Xylobiose	0.583 ± 0.039	n.a.*	*n.a*
Glucose	0.406 ± 0.160	*n.a*	*n.a*
Cellobiose	9.460 ± 0.608	1.300 ± 0.071	0.279 ± 0.008
Cellotriose	10.595 ± 0.246	3.767 ± 0.071	0.978 ± 0.022
Maltose	0.387 ± 0.104	*n.a*	*n.a*
Maltotriose	0.645 ± 0.010	*n.a*	*n.a*
No sugar	0.386 ± 0.034	*n.a*	*n.a*

^*^n.a. not analyzed (lack of biomass)

A distinct scenario arises when examining the metabolism of glucose in *C. thermodepolymerans*. The wild-type strain of this bacterium exhibits limited efficiency in metabolizing the monosaccharide. Surprisingly, the bacterium demonstrates remarkable proficiency in utilizing glucose oligomers linked by β−1,4-glycosidic bonds, such as cellobiose and cellotriose. Strikingly, the growth and synthesis of PHB on these glucose oligosaccharides, particularly cellotriose, surpass even that observed on the preferred substrate—xylose. However, the ability of *C. thermodepolymerans* to grow on substrates composed of glucose units by α−1,4-glycosidic linkages was very limited, indicating a clear preference for β-linked oligomers.

### Insight into the kinetics of utilization of xylose, glucose, and cellobiose

**Fig. 1:**
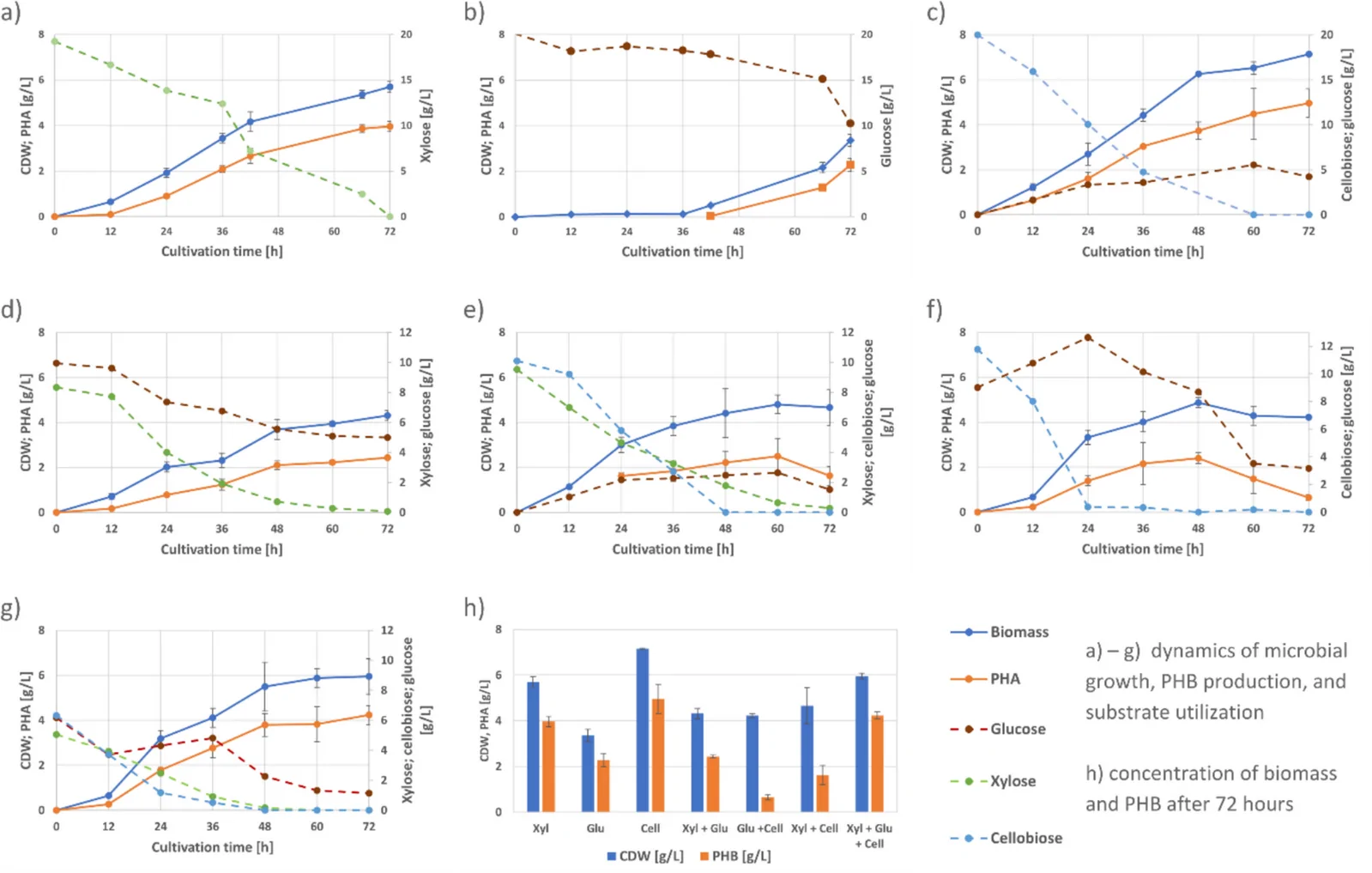
Growth of *C. thermodepolymerans* on xylose, glucose, cellobiose, and their binary and ternary mixtures. Cultivations were carried out in flasks in MSM mineral medium at 50 °C under constant shaking at 180 rpm in biological triplicates. The initial concentrations of the tested sugars were set at 20 g/L. In the case of binary and ternary mixtures, all sugars were applied in equal amounts. The samples were taken every 12 h and analyzed for CDW, PHB, and residual sugar contents. Used carbon substrates: **a** xylose, **b** glucose, **c** cellobiose, **d** xylose and glucose, **e** xylose and cellobiose, **f** glucose and cellobiose, **g** mixture of xylose, glucose, and cellobiose. **h** Comparison of biomass concentrations and PHB content after 72 h of cultivation on different carbon sources

To gain deeper insights into the sugar preference and metabolism of *C. thermodepolymerans*, we conducted a study on the kinetics of culture growth, PHB accumulation, and sugar consumption. The organism was cultivated on xylose, glucose, cellobiose, and various combinations of these sugars. In all cases of experimental set in Erlenmeyer flasks, the initial sugar concentration was set at 20 g/L. Since substrate concentrations were not normalized to total carbon content, the observed growth responses reflect substrate-specific metabolic utilization rather than direct comparisons of carbon yield. The results of these experiments are demonstrated in Table 2 and in Fig. 1.

**Table 2:** Kinetic parameters and yield coefficients describing the growth of *C. thermodepolymerans*on xylose, glucose, cellobiose, and their binary and ternary mixtures. Cultivations were carried out in flasks in MSM mineral medium at 50 °C under constant shaking at 180 rpm. The initial concentrations of the tested sugars were set at 20 g/L. In the case of binary and ternary mixtures, all sugars were applied in equal amounts

Substrate	*Y*_X/S_	*Y*_P/S_	Maximal volumetric biomass productivity (g/(Lh))	Maximal volumetric PHB productivity (g/(Lh))	Maximal sugar utilization rate (g/(Lh))		
					Xylose	Glucose	Cellobiose
Xyl	0.30	0.21	0.082	0.066	0.269	*n.a*	*n.a*
Glu	0.26	0.17	*0.084	0.068	*n.a*	0.252	*n.a*
Cel	0.44	0.31	0.140	0.074	*n.a*	*n.a*	**0.432
Xyl + Glu	0.35	0.20	0.060	0.040	0.152	0.090	*n.a*
Glu + Cel	0.24	0.04	0.111	0.060	*n.a*	0.213	0.475
Xyl + Cel	0.25	0.09	0.089	0.025	0.129	*n.a*	0.253
Xyl + Glu + Cel	0.36	0.26	0.129	0.096	0.107	0.145	0.132

^*^Lag-phase of 36 h in case of glucose

^**^Conversion of cellobiose into glucose, from 38% (12 h of cultivation) to 23% (36 h of cultivation)

*Y*_X/S_, *Y*_P/S_, yield coefficient; biomass (X), respectively product (P) yield on substrate (g/g)

When evaluating growth on the monosaccharides xylose and glucose, as expected, xylose proved to be a more suitable carbon source for the cultivation of *C. thermodepolymerans* (Fig. 1a, b). The final CDW value, PHB titer, and yield coefficients were significantly higher on xylose compared to glucose. Growth on glucose was associated with a prolonged lag phase (36 h), which led to lower overall efficiency in terms of PHB production. However, once the culture overcame the lag phase, the kinetic parameters—including maximal volumetric biomass or PHB productivity and sugar consumption rate (see Table 2)—observed on glucose were comparable to those obtained on xylose.

Interestingly, among the sugars tested in the flask experiment, the disaccharide cellobiose was found to be the most preferred. It supported the highest CDW and PHB titers. The culture also exhibited the greatest biomass and PHB productivity and the most efficient sugar consumption. However, even the utilization of cellobiose revealed some problematic aspects. During cultivation, glucose was detected in the culture medium, suggesting that a portion of cellobiose underwent hydrolysis (Fig. 1c). For instance, approximately 38% of cellobiose consumed during the first 12 h of cultivation was extracellularly converted into glucose, and 23% was similarly converted at 36 h.

Nevertheless, the extracellular cleavage of cellobiose into glucose appears to be a disadvantage for efficient cellobiose utilization. Due to the low efficiency of glucose metabolism in *C. thermodepolymerans*, extracellularly cleaved glucose accumulates in the medium, with residual concentrations reaching 4.22 g/L after 72 h of cultivation (Fig. 1b). This inefficiency suggests that the overall utilization of cellobiose could be significantly improved if extracellular cleavage was minimized or even eliminated. On the other hand, the ability of *C. thermodepolymerans* to efficiently utilize cellobiose suggests that in this bacterium, the challenges associated with glucose assimilation are more likely related to deficiencies in glucose transport into the cell rather than limitations in its intracellular catabolic pathways.

### Growth, PHB production, and sugars utilization kinetics on sugar mixtures

In addition to single sugars, we also conducted a study on binary (Fig. 1d, e, f) and ternary (Fig. 1g) mixtures of glucose, xylose, and cellobiose. All the tested sugars were applied at equal concentrations, with an initial total sugar concentration of 20 g/L.

The utilization of sugar mixtures, particularly binary sugar mixtures, generally resulted in lower culture growth efficiency and, most notably, reduced PHB accumulation compared to cultivation on single sugars, such as xylose or cellobiose. The cultivations using sugar mixtures yielded lower PHB titers and a reduced PHB content in bacterial biomass (see Fig. 1h).

When glucose was combined with xylose (Fig. 1d), as expected, xylose was consumed at a significantly higher rate and was completely depleted during cultivation. In contrast, glucose was utilized at a considerably lower rate, with approximately 50% of its initial concentration remaining unconsumed. This clearly demonstrates the preferential uptake of xylose over glucose by *C. thermodepolymerans*. However, this preference is most likely not driven by a carbon catabolite repression mechanism (Görke and Stülke 2008), as no diauxia was observed and both substrates were co-consumed throughout the entire cultivation.

Surprisingly, even the combination of xylose and cellobiose (Fig. 1e), two sugars that are efficiently utilized when supplied individually, resulted in a low *Y*_P/S_ coefficient and reduced PHB titers. During cultivation, the consumption rate of cellobiose (0.253 g/(L·h)) was nearly twice that of xylose (0.129 g/(L·h)). However, a significant portion of the consumed cellobiose underwent extracellular cleavage. For instance, after 12 h of cultivation, nearly all the utilized cellobiose had been converted into glucose, and by 24 h, the cellobiose-to-glucose conversion rate reached 46%. Notably, the resulting glucose remained unutilized in the culture medium. This indicates that the extracellular cleavage of cellobiose, particularly pronounced in the presence of xylose, negatively impacted the efficiency of PHB production by *C. thermodepolymerans* when cultivated on a xylose-cellobiose mixture.

Furthermore, cultivation on a glucose-cellobiose mixture resulted in the lowest PHB yield and titer among all tested cultures (Fig. 1f). Once again, cellobiose was consumed at a very high rate (0.475 g/(L·h), the highest observed in this study). However, due to partial extracellular cleavage of cellobiose, an increase in glucose concentration was observed in the cultivation medium during the initial 24 h. As cellobiose was completely depleted during the initial 24 h, the microbial culture subsequently began utilizing the accumulated glucose. Nevertheless, as previously noted, the overall productivity of the system remained very low.

Last but not least, the cells were also cultivated in a ternary mixture containing all three sugars—glucose, xylose, and cellobiose (Fig. 1g). Compared to binary mixtures, the PHB yields, PHB titers, and PHB content in biomass were significantly higher and nearly comparable to those obtained with single xylose or cellobiose as the carbon source. Once again, cellobiose was consumed at a higher rate than xylose, and its metabolism was associated with glucose formation. This effect was particularly evident between 12 and 36 h of cultivation when an increase in glucose concentration was detected. By 48 h, both xylose and cellobiose were fully utilized, and even the residual glucose concentration remained very low (only about 1.14 g/L) compared to other cultivation scenarios tested in this experiment.

### Integration of exogenous glucose transporter from* Z. mobilis *into genome of *C. thermodepolymerans*

**Fig. 2:**
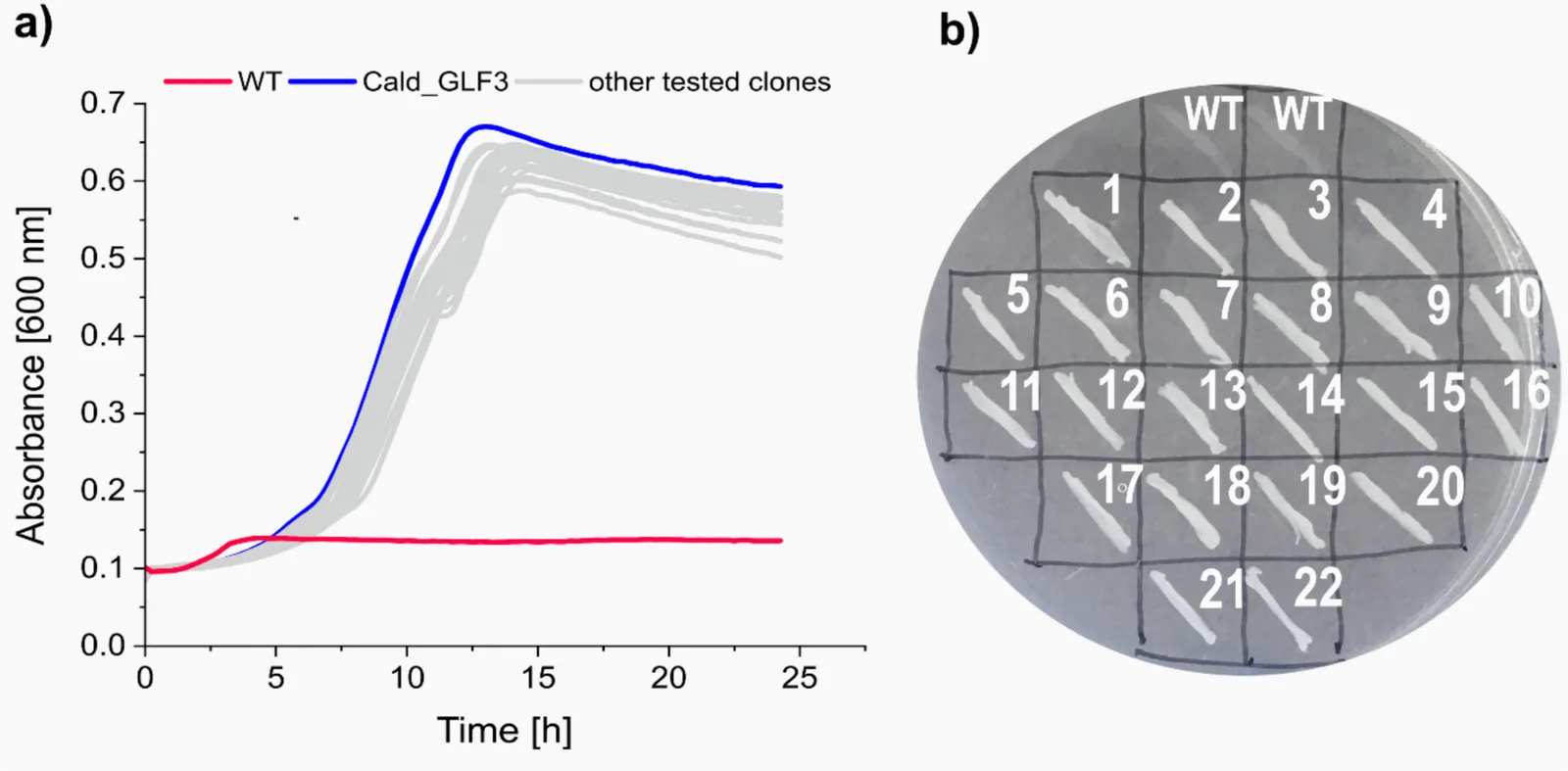
Growth of *C. thermodepolymerans* clones isolated after the integration of the *glf* glucose facilitator gene into the chromosome on glucose. **a** Growth of *C. thermodepolymerans* isolates with integrated *glf* and wild-type control in MSM minimal medium with 2 g/L glucose and 10 µg/mL gentamicin in a 48-well plate at 42 °C. **b***C. thermodepolymerans* wild type and isolated clones 1–22 with integrated *glf* grown on MSM plates supplied with 5 g/L glucose and 10 µg/mL gentamicin at 42 °C

To test the hypothesis on the glucose transport bottleneck, we endowed *C. thermodepolymerans* with well-characterized glucose facilitator Glf from *Z. mobilis* (Parker et al. 1995). The *glf* gene with consensus Shine-Dalgarno sequence AGGAGG was randomly integrated into the *C. thermodepolymerans* chromosome via the mini-Tn5 transposon vector pBAMD1-6 (Martínez-García et al. 2014) allowing for chromosome positioning effects and the selection of transformants with balanced *glf* expression and reduced metabolic burden from exogenous genetic material (Demko et al. 2019). The introduction of the vector into the cells was achieved through electroporation, followed by selection in minimal medium with glucose at 42 °C. We observed none or negligible growth of transformants at 50 °C. However, the selection at 42 °C resulted in visible growth of the library of transformants in liquid medium with glucose. Individual clones growing fast on glucose were isolated on agar plates. Forty isolated clones were tested for growth on glucose as a sole carbon and energy source in a 48-well plate format and on agar plates (Fig. 2). Cald_GLF3 with confirmed *glf* integration in the intergenic region between the GMC family oxidoreductase (locus tag IS481_17915) and the hypothetical protein (locus tag IS481_17920) in chromosome emerged as the most promising candidate for further characterization.

**Fig. 3:**
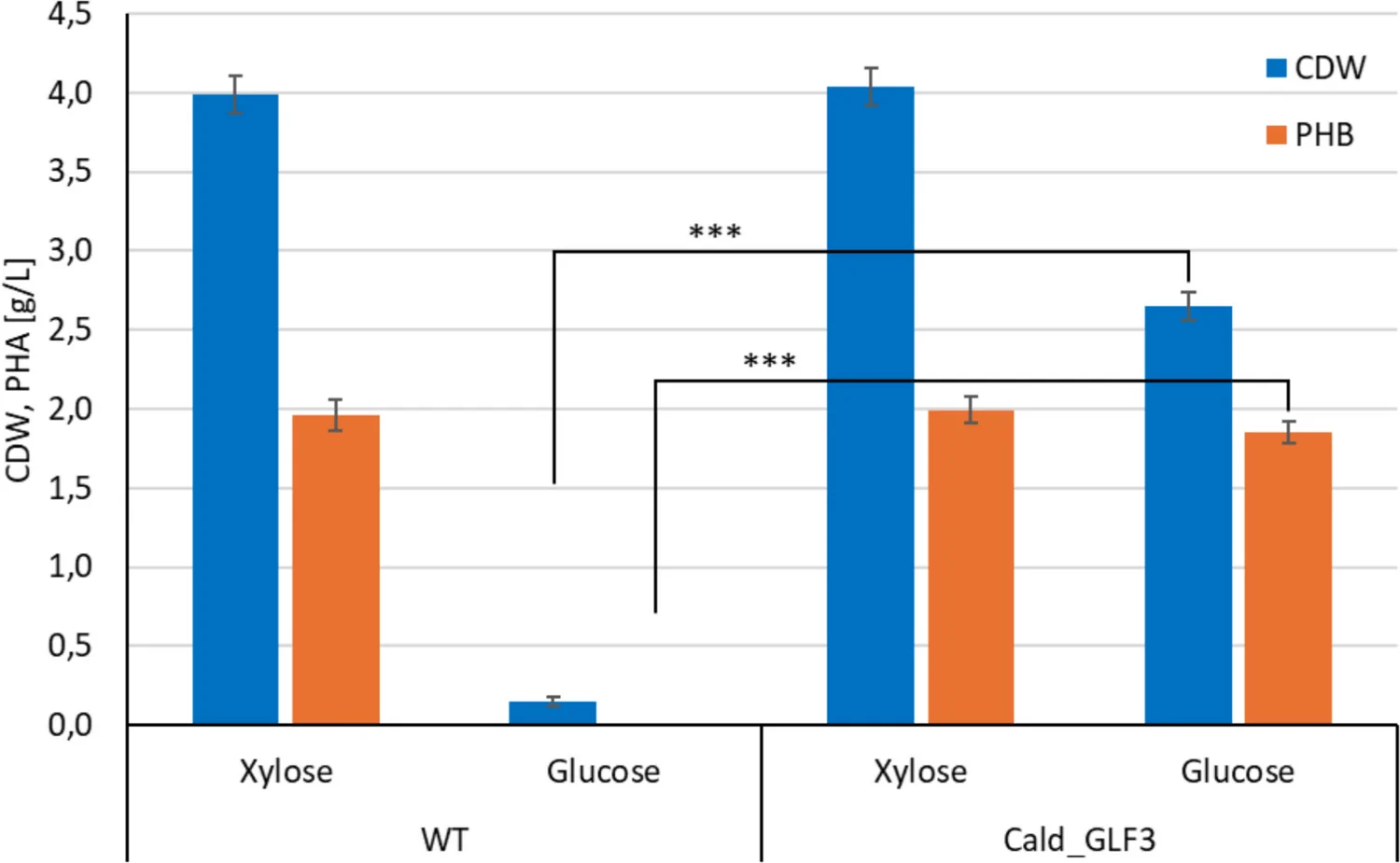
Growth and PHB production capacity of *C. thermodepolymerans*—wild type and strain Cald_GLF3 harboring the glucose transporter from *Z. mobilis*—on xylose and glucose. Cultivations were carried out in biological triplicates in flasks with MSM mineral medium at 42 °C (a suboptimal temperature for *C. thermodepolymerans*) due to the temperature instability of the Glf transporter. The initial sugar concentration was set at 20 g/L, and cultivations were performed under constant shaking (180 rpm) for 72 h. Data represent mean ± SD of biological triplicates (*n* = 3). Statistical significance was determined using an unpaired Student’s *t*-test. ***p < 0.001

Further, we tested the PHB production performance of the Cald_GLF3 strain and compared it to the wild type when cultivated on xylose and glucose. The results are presented in Fig. 3. It should be noted that this experiment was conducted at 42 °C due to the thermal instability of the Glf transporter. However, this temperature is suboptimal for the cultivation of *C. thermodepolymerans*, which explains the lower PHB and biomass yields compared to those observed in the wild type cultivated at 50 °C (see Fig. 1 and Table 2). Interestingly, the growth and PHB production capacity of Cald_GLF3 on xylose remained unchanged compared to the WT. However, as expected, the introduction of the Glf transporter significantly improved growth on glucose. Although a direct comparison of the WT strain cultivated on glucose at 50 °C with the engineered Cald_GLF3 strain cultivated on glucose at 42 °C revealed a modest reduction in biomass and PHA production (approximately 20%), the WT strain exhibited severe growth impairment at 42 °C. In contrast, the engineered Cald_GLF3 strain maintained robust growth and PHA production at this temperature, resulting in ~ 17-fold increase in biomass and enabling PHA synthesis under conditions where the WT strain failed to grow. Statistical analysis confirmed that both CDW and PHB titers of Cald_GLF3 on glucose were significantly higher than those of the wild-type strain (*p* < 0.001). Conversely, no statistically significant difference in growth or PHB production was observed between the wild-type strain and Cald_GLF3 when cultivated on xylose at 42 °C (*p* > 0.05). While the wild type exhibited only minimal growth on glucose at 42 °C, the Cald_GLF3 strain demonstrated robust growth without a noticeable lag phase. Moreover, the PHB titer obtained on glucose was comparable to that observed for both the wild type and Cald_GLF3 on xylose.

Motivated by the successful PHB production of the Cald_GLF3 strain on glucose, we decided to further investigate its sugar-utilization capacity. Therefore, similar to the wild-type strain (Table 2, Fig. 1), we cultivated Cald_GLF3 in the presence of xylose, glucose, cellobiose, and their binary and ternary mixtures. Again, the cultivation of Cald_GLF3 was carried out at 42 °C. In addition to measuring CDW and PHB yields, we also recorded the concentrations of residual sugars, and the results are summarized in Table 3. As previously mentioned, the suboptimal cultivation temperature resulted in lower CDW values for Cald_GLF3 cultures grown on glucose and xylose compared to the wild-type strain cultivated at 50 °C. Interestingly, we observed relatively high amounts of residual monosaccharides after cultivation, which can likely be attributed to less efficient metabolism at a lower temperature. However, the results for cellobiose were surprisingly different. The Cald_GLF3 strain was able to almost completely utilize cellobiose, achieving the highest CDW and PHB titers observed in our flask cultivations so far—11.5 g/L and 9.6 g/L, respectively.

**Table 3:** Growth, PHB production, and sugar consumption of Cald_GLF3 strain cultivated on xylose, glucose, cellobiose, and their binary and ternary mixtures. Cultivations were carried out in biological triplicates in flasks with MSM mineral medium at 42 °C under constant shaking at 180 rpm, 72 h. The initial concentrations of the tested sugars were set at 20 g/L. In the case of binary and ternary mixtures, all sugars were applied in equal amounts

	CDW (g/L)	PHB g/L (g/L)	PHB % (g/L)	Residual saccharide (g/L)			Yield coefficients	
				Xyl	Glu	Cel	***Y***_X/S_	***Y***_P/S_
Xyl	4.04 ± 0.12	1.99 ± 0.08	49.33 ± 2.07	12.79 ± 2.35	*n.d*	*n.d*	0.56	0.28
Glu	2.65 ± 0.09	1.85 ± 0.07	69.84 ± 2.52	*n.d*	15.92 ± 1.85	*n.d*	0.65	0.45
Cel	11.49 ± 0.22	9.62 ± 0.33	83.74 ± 2.40	*n.d*	0.82 ± 0.07	*n.d*	0.60	0.50
Xyl + glu	3.14 ± 0.17	2.30 ± 0.14	73.17 ± 1.52	10.95 ± 0.75	3.17 ± 0.98	*n.d*	0.53	0.39
Glu + cel	2.70 ± 0.42	1.83 ± 0.31	67.66 ± 2.54	*n.d*	6.57 ± 0.40	9.20 ± 0.31	0.64	0.43
Xyl + cel	7.66 ± 0.40	5.61 ± 0.27	73.33 ± 1.69	0.25 ± 0.01	0.75 ± 0.01	5.25 ± 0.72	0.56	0.41
Xyl + glu + cel	5.52 ± 0.19	3.83 ± 0.22	69.40 ± 2.30	4.18 ± 1.05	1.70 ± 0.92	0.20 ± 0.34	0.41	0.28

In the case of saccharide mixtures, the introduction of *glf* from *Z. mobilis* into the genome of *C. thermodepolymerans* had a notably positive effect, especially in the presence of cellobiose (Table 3). Despite being cultivated at a suboptimal temperature, the Cald_GLF3 strain demonstrated higher PHB productivity than the wild-type strain (Table 2). This effect was particularly evident in the binary mixture of xylose and cellobiose, which resulted in exceptionally high CDW and PHB titers (7.66 g/L and 5.61 g/L, respectively). Similarly, the ternary mixture of the tested saccharides yielded promising results. Conversely, the mixture of glucose and cellobiose was the least efficient. Notably, a significant portion of cellobiose (92% of the initial amount) remained unconsumed in the cultivation medium. This combination was also the least productive for the wild-type strain (Table 2). However, in this case, the Cald_GLF3 strain exhibited substantially higher PHB productivity (1.83 g/L) compared to the wild-type strain (0.64 g/L), highlighting the potential of engineered *C. thermodepolymerans* for PHB production from plant-derived sugars. Results from cultivations with glucose and xylose, or mixtures of glucose, xylose, and cellobiose, indicate a reversed sugar preference in the Cald_GLF3 strain compared to the wild type.

## Discussion

**Fig. 4:**
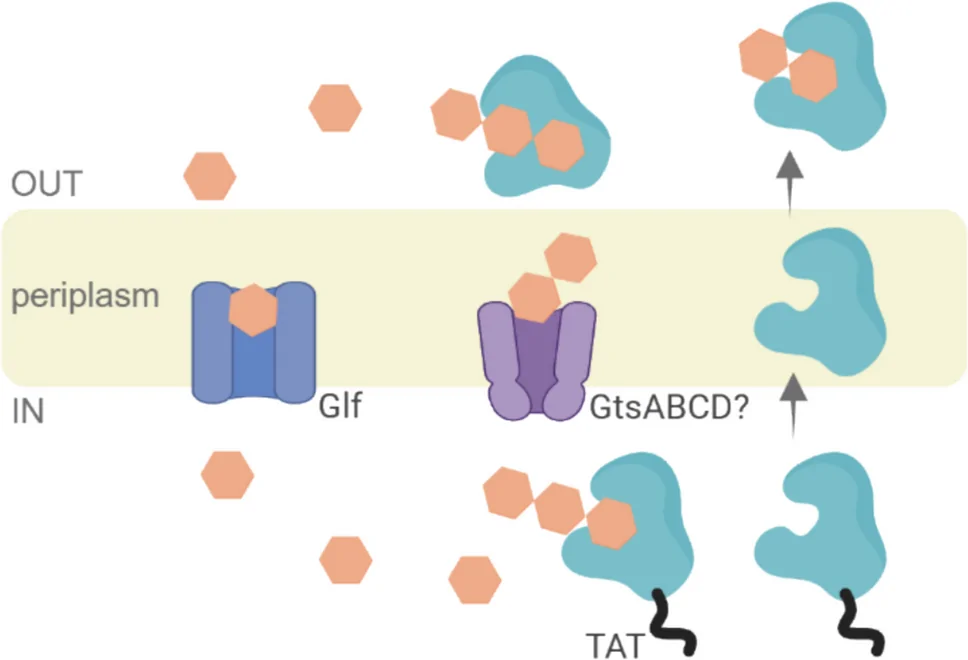
Simplified scheme of proposed glucose and cellooligosaccharide transport in engineered *C.thermodepolymerans* strain Cald_GLF3. β-glucosidase with or without a TAT signal peptide is shown in cyan; beige hexagons represent monomeric glucose or its dimeric and trimeric forms, cellobiose and cellotriose, respectively. Individual components of the scheme are not to scale. Created with BioRender.com

Consistent with prior findings (Kourilova et al. 2020; Musilova et al. 2023), *C. thermodepolymerans* utilizes xylose significantly more efficiently than other monosaccharides, especially glucose. This holds significant importance as xylose stands out as the most abundant pentose and, after glucose, the second most prevalent monosaccharide in lignocellulosic biomass. The efficient conversion of xylose into valuable chemicals, fuels, and materials emerges as a critical task for establishing a sustainable bioeconomy (Narisetty et al. 2022). In this light, due to its high affinity to xylose, *C. thermodepolymerans* appears to be a distinctive bacterium, presenting a promising candidate as an NGIB chassis for valorising xylose-rich resources. Despite the markedly lower biomass yield observed on glucose compared to xylose, it is intriguing that *C. thermodepolymerans* exhibited a high capacity to metabolize β−1,4-linked glucose oligomers such as cellobiose and cellotriose. The well assay revealed the highest growth and production on cellotriose. This suggests that the bacterium may possess specialized transport and enzymatic systems enabling efficient utilization of these oligomeric substrates. Cellotriose preference may reflect a potential energetic advantage of transporting longer oligosaccharides compared to shorter ones like cellobiose or monomeric glucose. If sugars are actively transported through one of the present ABC transporters (possibly a GtsABCD glucose/mannose transporter present in DSM 15344 with loci IDs IS481 00940, IS481 00945, IS481 00950, IS481 00955) and intracellular cleavage of cellooligosaccharides occurs via previously identified β-glucosidase (gene locus ID481 00935) (Musilova et al. 2023), only one ATP molecule is required per cellotriose, yielding three glucose molecules (Fig. 4). In contrast, cellobiose yields only two glucose molecules for the same energy cost (Dvořák and de Lorenzo 2018; Parisutham et al. 2017). ATP-dependent cellooligosaccharide ABC transporters and β-glucosidases were found in diverse thermophilic bacteria such as *Pyrococcus*, *Clostridium*, *Thermotoga*, or *Thermococcus* (Tjo and Conway 2024), and bioenergetic advantage of preferential uptake of longer cellodextrins was confirmed, e.g., for *C. thermocellum* (Tjo and Conway 2024; Zhang and Lynd 2005).

In contrast, the presence of α−1,4-glycosidic bonds, as found in maltose or maltotriose, impedes the metabolization of these glucose oligosaccharides by *C. thermodepolymerans*. The inability to cleave α−1,4-glycosidic bonds renders the wild-type strain of *C. thermodepolymerans* unsuitable as a PHB producer from starch and similar saccharides. However, it is worth noting that other members of the *Caldimonas* genus, such as *C. taiwanensis* (Chen et al. 2005; Sheu et al. 2009), possess amylolytic activity. Therefore, they could serve as suitable sources for genes that could enhance the biotechnological potential of *C. thermodepolymerans* through metabolic engineering techniques.

Analysis of substrate utilization kinetics indicates the culture requires an adaptation period for efficient growth on glucose, likely involving the activation of specific metabolic processes. Examination of the kinetics for other substrates and their combinations further supported the previously discussed findings that the metabolism of oligosaccharides is often more efficient than that of monosaccharides (Dvořák and de Lorenzo 2018). However, even the utilization of cellobiose revealed some problematic aspects. During cultivation, glucose was detected in the culture medium, suggesting that a portion of cellobiose underwent hydrolysis. Two potential mechanisms may explain this phenomenon. The first hypothesis is that cellobiose is transported into the cells, hydrolyzed to glucose intracellularly, and subsequently excreted before its phosphorylation by glucokinase Glk. However, this scenario is very unlikely, as cells rarely excrete valuable metabolites like hexoses, and phosphorylation is a rapid step. A more plausible explanation is that *C. thermodepolymerans* possesses enzymatic machinery capable of both intracellular and extracellular cleavage of cellobiose (Fig. 4). Notably, a signal sequence specific to the twin-arginine translocation (Tat) pathway was identified at the 5′ end of the β-glucosidase gene IS481 00935 (Musilova et al. 2023). Since the Tat pathway is known to translocate fully folded and active proteins across the cytoplasmic membrane (Berks et al. 2003), it is hypothesized that the enzyme hydrolyzes cellobiose during its passage from the cell, in the cytoplasm, periplasm, and the surrounding medium (Fig. 4). On the other hand, the ability of *C. thermodepolymerans* to efficiently utilize cellobiose suggests that in this bacterium, the challenges associated with glucose assimilation are more likely related to deficiencies in glucose transport into the cell rather than limitations in its intracellular catabolic pathways.

Generally, it is evident that the biotechnological potential of *C. thermodepolymerans* in metabolizing lignocellulose-derived sugars is significantly constrained by its inefficient glucose utilization and the partial extracellular hydrolysis of cellobiose into glucose. Both of these phenomena have a substantial negative impact on the productivity of the microbial culture, particularly in binary mixtures of the investigated sugars. However, as mentioned above, since the bacterial culture efficiently utilizes cellobiose, we hypothesize that the issue with glucose utilization is primarily due to inefficient glucose transport into the bacterial cell, rather than a deficiency in the metabolic pathways required for glucose catabolism. Therefore, equipping *C. thermodepolymerans* with a highly efficient glucose transporter could potentially resolve both issues; it would enhance glucose uptake and utilization, thereby mitigating also the negative effects associated with partial extracellular hydrolysis of cellobiose into glucose.

To verify this hypothesis, we integrated an exogenous glucose transporter from the bacterium *Z. mobilis* into the genome of wild-type *C. thermodepolymerans*. The Glf protein is the sole known bacterial glucose facilitator within the major facilitator superfamily (MFS) that operates as a sugar uniporter, enabling facilitated diffusion of monosaccharides (Kurgan et al. 2022). Given its passive transport mechanism, Glf is an extremely efficient energy-saving alternative to other substrate uptake systems from among bacterial MFS that consume ATP or employ concurrent proton transfer. Although different thermophilic transport systems have been reported in the literature, sugar transport in thermophiles is still poorly understood and investigated (Tjo and Conway 2024), and we are not aware of any characterized thermophilic homologue of Glf. Therefore, we settled for the mesophilic Glf in this study and tested its function in *C. thermodepolymerans*. Our findings suggest that equipping the strain with a potent glucose facilitator effectively resolved limitations associated with cellobiose metabolism, particularly the extracellular cleavage of cellobiose into glucose, which was most likely quickly and efficiently transferred to the cells and metabolized. However, we must acknowledge that the exceptionally high productivity of Cald_GLF3 on cellobiose exceeded our expectations, and we currently lack a definitive explanation for this remarkable result. We hypothesize that the introduction of a new glucose transporter may have also enhanced the activity or expression of the native cellobiose transporter. This potential crosstalk between sugar transport systems could have resulted in highly efficient sugar uptake and subsequent metabolism. Alternatively, it can be speculated that the Glf facilitator of *Z. mobilis* may exhibit broader substrate specificity (especially at elevated temperatures), potentially facilitating the transport of cellobiose as well. This speculation aligns with the general understanding that some sugar transporters, particularly those in fungi and bacteria, can transport a variety of sugars, including disaccharides, due to their low substrate specificity (Barbi et al. 2021). Undoubtedly, the observed improvement in cellobiose utilization by Cald_GLF3 is a fascinating phenomenon that deserves further investigation. The shift in sugar preference from xylose to glucose in glucose–xylose mixtures observed for the Cald_GLF3 strain (Table 3), compared with the wild type (Fig. 1), is also intriguing and is currently the subject of a detailed investigation in our ongoing research. Thermophilic microorganisms reported in the literature produce PHA at moderate titers under flask batch cultivation conditions, with concentrations often below 2 g/L for *Aneurinibacillus* (Pernicova et al. 2020; Xiao et al. 2015) and *Bacillus* species (Choonut et al. 2020; Liu et al. 2014) cultivated at 45–55 °C. Higher PHB titers have been reported for some *Chelatococcus* strains (Ibrahim et al. 2016; Xu et al. 2014) grown on glucose at 50–55 °C, reaching up to 16.8 g/L under optimized conditions in bioreactor (Ibrahim and Steinbüchel 2010). In comparison, Cald_GLF3 developed in this study produced up to 9.6 g/L PHB from cellobiose at 42 °C in shake-flask cultivation, which is comparable to or higher than values reported for many thermophilic PHA producers under similar batch conditions.

Taken together, these results confirm our hypothesis and show that *C. thermodepolymerans* DSM 15344 can use glucose monomer as the sole carbon and energy source for growth and PHB biosynthesis once the transport bottleneck is removed, e.g., by implantation of an exogenous glucose facilitator. It should be noted that this is not yet an ideal solution as the temperature optimum of the glucose transporter used does not match the growth optimum of *C. thermodepolymerans*. This temperature incompatibility can be overcome by protein engineering and stabilization of the Glf transporter (Dvorak et al. 2017; Musil et al. 2023) or by searching for a suitable thermostable homologue in databases (Hon et al. 2020). Alternatively, faster growth of DSM 15344 on glucose can be achieved by laboratory evolution (Espeso et al. 2020). We plan to investigate these options in our further work.

## Conclusion

*C. thermodepolymerans* DSM 15344 exhibits a distinctive utilization profile for lignocellulose-derived sugars. Despite its limited native capacity for glucose assimilation, primarily due to a transport bottleneck, the bacterium shows remarkable potential for PHA production from selected plant-relevant carbohydrates. The observed extracellular cleavage of cellobiose into glucose, coupled with inefficient glucose uptake, significantly constrains bioprocess efficiency, particularly in sugar mixtures. Introducing of the *glf* gene from *Z. mobilis* successfully removed this transport limitation, enabling robust glucose metabolism and markedly improving cellobiose utilization. However, due to the mesophilic origin of Glf, the engineered strain was operated at 42 °C, below the natural growth optimum of *C. thermodepolymerans*. Future work should therefore focus on stabilizing the Glf transporter to withstand higher cultivation temperatures or on identifying alternative thermostable glucose transporters, allowing growth at 50°C. These improvements, alongside broader transporter engineering and adaptive laboratory evolution, could unlock the full potential of *C. thermodepolymerans* as an NGIB chassis for efficient lignocellulose valorisation into sustainable bioplastics.

## Data Availability

The data are available from the corresponding author upon reasonable request.
